# Identification of Immune Signatures of Novel Adjuvant Formulations Using Machine Learning

**DOI:** 10.1038/s41598-018-35452-x

**Published:** 2018-11-30

**Authors:** Sidhartha Chaudhury, Elizabeth H. Duncan, Tanmaya Atre, Casey K. Storme, Kevin Beck, Stephen A. Kaba, David E. Lanar, Elke S. Bergmann-Leitner

**Affiliations:** 1 Biotechnology High Performance Computing Software Applications Institute, Telemedicine and Advanced Technology Research Center, U.S. Army Medical Research and Materiel Command, Fort Detrick, MD USA; 2 Malaria Vaccine Branch, US Military Malaria Research Program, Walter Reed Army Institute of Research, Silver Spring, MD USA; 3 Miltenyi Biotec Inc., San Diego, CA USA

## Abstract

Adjuvants have long been critical components of vaccines, but the exact mechanisms of their action and precisely how they alter or enhance vaccine-induced immune responses are often unclear. In this study, we used broad immunoprofiling of antibody, cellular, and cytokine responses, combined with data integration and machine learning to gain insight into the impact of different adjuvant formulations on vaccine-induced immune responses. A Self-Assembling Protein Nanoparticles (SAPN) presenting the malarial circumsporozoite protein (CSP) was used as a model vaccine, adjuvanted with three different liposomal formulations: liposome plus Alum (ALFA), liposome plus QS21 (ALFQ), and both (ALFQA). Using a computational approach to integrate the immunoprofiling data, we identified distinct vaccine-induced immune responses and developed a multivariate model that could predict the adjuvant condition from immune response data alone with 92% accuracy (p = 0.003). The data integration also revealed that commonly used readouts (i.e. serology, frequency of T cells producing IFN-γ, IL2, TNFα) missed important differences between adjuvants. In summary, broad immune-profiling in combination with machine learning methods enabled the reliable and clear definition of immune signatures for different adjuvant formulations, providing a means for quantitatively characterizing the complex roles that adjuvants can play in vaccine-induced immunity. The approach described here provides a powerful tool for identifying potential immune correlates of protection, a prerequisite for the rational pairing of vaccines candidates and adjuvants.

## Introduction

Adjuvants have long been recognized as a crucial component in vaccine formulations^1^. Currently, access to, and availability of, adjuvants suitable for clinical use are limited; licensed vaccines are typically formulated with Alum, and there are only a few licensed novel vaccines, such as AS04 or AS01B. In an effort to develop vaccines without the need to depend on commercial partners, our institute has developed several liposomal adjuvant formulations^2–4^. These formulations contain liposomes as carriers for adjuvants monophospholipid A (MPLA) and either Alum and/or QS21). Liposomes containing MPLA induce high antibody titers as well as strong cellular responses^5^. Formulations of circumsporozoite protein (CSP) with QS21 have been used for the leading malaria vaccine RTS,S, which provides up to 83% vaccine efficacy depending on the vaccination regimen^6^. In the field, however, this formulation only provides limited and short-lived efficacy^7^. Therefore, new approaches to increase vaccine efficacy are needed.

Recently, a self-assembling protein nanoparticle (SAPN) platform displaying the C-terminus (denoted here as peptide 16 (PF16)), as well as six copies of the central repeat region motif of *Plasmodium falciparum* CSP (PfCSP), was manufactured for testing in humans^4^. This construct also contains two universal CD4^+^ T cell helper epitopes, derived from the Lymphocytic Choriomeningitis virus and the Influenza A matrix protein 1, to increase the immunogenicity of the SAPN. This SAPN, formulated with the commercially available analogue of MF59 (AddaVax), demonstrated the ability to mediate sterile protection in mice after challenge with a *P. berghei* transgenic parasite that expresses *Pf*CSP^8^, providing support for the vaccine’s clinical development. The mechanism of protection in mice was found to be based, at least in part, on antibody-mediated sporozoite neutralization. Although the role of cellular responses has not yet been fully explored, SAPNs have been shown to be taken up and processed by dendritic cells, suggesting the possibility of a role for cellular immunity in protection.

Cellular immunity is reportedly important for protection in the RTS,S malaria vaccine, another subunit vaccine based on the CSP antigen. Previous studies have shown that protection in RTS,S depends, at least in part, on CD4^+^ T cells, especially those producing IFN-γ, IL2, TNFα^9^, and antibodies (especially, certain isotype classes)^10,11^. Although induction of CD8^+^ responses in humans has proven difficult when immunizing with recombinant vaccines, such as RTS,S, DNA-based vaccines, such as the heterologous prime-boost DNA/Ad4 CSP-based vaccine, have demonstrated that CSP-specific CD8^+^ T cells can be induced. Likewise, a study using humanized mice demonstrated that CD8^+^ T cells can mediate protection when RTS,S is delivered with the CAF09 liposomal adjuvant^12^.

CD8^+^ T cells may increase the efficacy of a CSP-based vaccine because they could potentially eliminate *Plasmodium*-infected hepatocytes. Such an effect would complement antibody-based neutralization. While it is clear that the appropriate selection of adjuvant and vaccine delivery platform may be able to induce the desired cellular immunity, there are several challenges to identifying the correct vaccine formulation is challenging. First, adjuvants can induce a wide range of complex and subtle changes to vaccine-induced immunity. Quantification of these adjuvant effects can help guide the selection of adjuvants to achieve the desired mode of immunity. Second, experimental assessment of cellular immunity is complicated by the fact that certain cell types may migrate out of the blood compartment and into other organs, such as the liver, lymph node, or spleen, where they could be difficult to detect^13^.

In this study, we measure a wide range of serological, cellular, and cytokine properties in tissue samples taken from multiple physiological compartments, including the blood, liver, spleen, and lymph node, to generate a broad immunoprofile of non-human primates (NHPs) following vaccination with different SAPN/adjuvant formulations consisting of Alum, QS21, or a combination of both. The goal of the study was to determine the potency of different adjuvant formulations in a primate preclinical model. The resulting immunoprofile consisted of over 120 immune measures collected for each animal in the study. Machine-learning methods, such as the random forest model, offer powerful tools for analyzing large immune data sets and for characterizing how different immune features or combinations of immune features are linked to vaccination conditions or immunological outcomes^14–16^. This is particularly useful in immunology studies, where group-level or population-level effect sizes can be modest, and where sample sizes are relatively small compared to the number of immune parameters being measured^16^. We employed a range of multivariate analysis techniques to characterize immune responses with respect to adjuvant formulation and antigen dose, and thereby define a distinct immune ‘signature’ for each adjuvant and adjuvant/SAPN dosage combination used in the study.

Immune correlates – or even surrogates – of protection continue to remain elusive for most diseases, including malaria. Efforts to define immune parameters responsible for protection against infection or disease have been hampered by a focus on a limited number of “standard” immune parameters, most commonly serum antibody titers and/or the secretion of certain cytokines by antigen-specific T cells in select immune compartments (in case of NHP or humans, most commonly PBMC). While the choice of adjuvant can determine whether or not a vaccine is capable of inducing protective immunity by altering a vaccine’s immune profile, adjuvants continue to be selected based on factors such as availability, since rational adjuvant-vaccine pairing requires insights into both immune correlates of protection and immunoprofiles induced by adjuvants. The study presented here represents a first step towards this goal by providing the tools, namely laboratory assays, statistical methods, and mathematical models, capable of identifying characteristic immune profiles imprinted upon a vaccine by different adjuvant formulations.

## Results

### Vaccinations with SAPN

Vaccinations were carried out in 22 rhesus monkeys (*Macaca mulatta*) randomly assigned to nine cohorts (Supplemental Table S1). Each vaccine and adjuvant cohort consisted of three animals, except the control cohorts which each had a single animal. Three different adjuvant conditions were compared side-by-side: ALFA, ALFQ, and the combination ALFQA formulation. The SAPN-based vaccine (FMP014) was administered at two antigen doses: 10 µg (“low dose”) and 40 µg (“high dose”), for the low-dose and high-dose cohorts, respectively. The immunization schedule for both vaccinated and control animals was three intramuscular injections at four week intervals. Because of the relatively small sample sizes in each cohort, we pooled vaccinated subjects by adjuvant (ALFA, ALFQ, and ALFQA) and antigen dose (low dose and high dose). For comparison, we also pooled the adjuvant-only and saline conditions as a combined ‘non-vaccinated’ group. Although this sample size is relatively small, it is typical for many NHP studies. In order to apply more sophisticated data analysis methods such as machine-learning methods and regression analysis on this limited sample size, we employed a number of strategies including feature reduction and boot-strapping to minimize the risk of overfitting and assess the variability in the model estimates.

### Immunoprofiling

**Figure 1:**
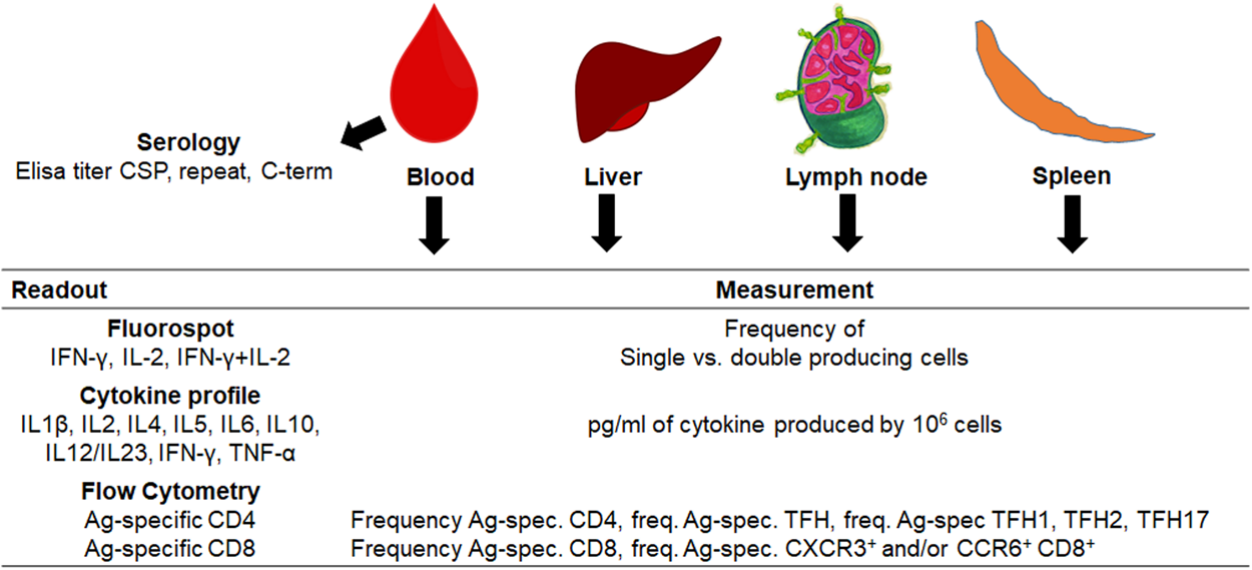
Overview of all measurements collected in this study. Samples were collected from blood, liver, lymph node, and spleen. Serology, Fluorospot, cytokine, and flow cytometry assays were carried out for all tissues and different time points for peripheral blood mononuclear cells (PBMCs).

Our profiling panel includes serological tests (i.e. ELISA titers to the CSP-SAPN, the repeat region, and the C-terminus of CSP), cellular assays measuring the frequency of CD4^+^ and CD8^+^ T cell subpopulations, as well as arrays profiling the cytokine response in antigen-stimulated leukocytes (Fig. 1).

Vaccine-induced responses were monitored by bleeding the animals prior to the start of the study and after each vaccination. Sera from these time points were used to determine the potency of the vaccine formulations. IFN-γ/IL2 Fluorospot assays were used to measure changes in the frequency of CSP-specific cells between pre-immune, post-vaccination, and terminal time points. After completing the vaccination regimen, animals were euthanized and blood, livers, draining lymph nodes, and spleens were collected. Cryopreserved leukocytes from the various compartments were either unstimulated or stimulated with CSP-SAPN or CSP peptide pools. Animals receiving saline/adjuvant formulations were used to determine vaccine-specific responses.

### Hierarchical clustering

**Figure 2:**
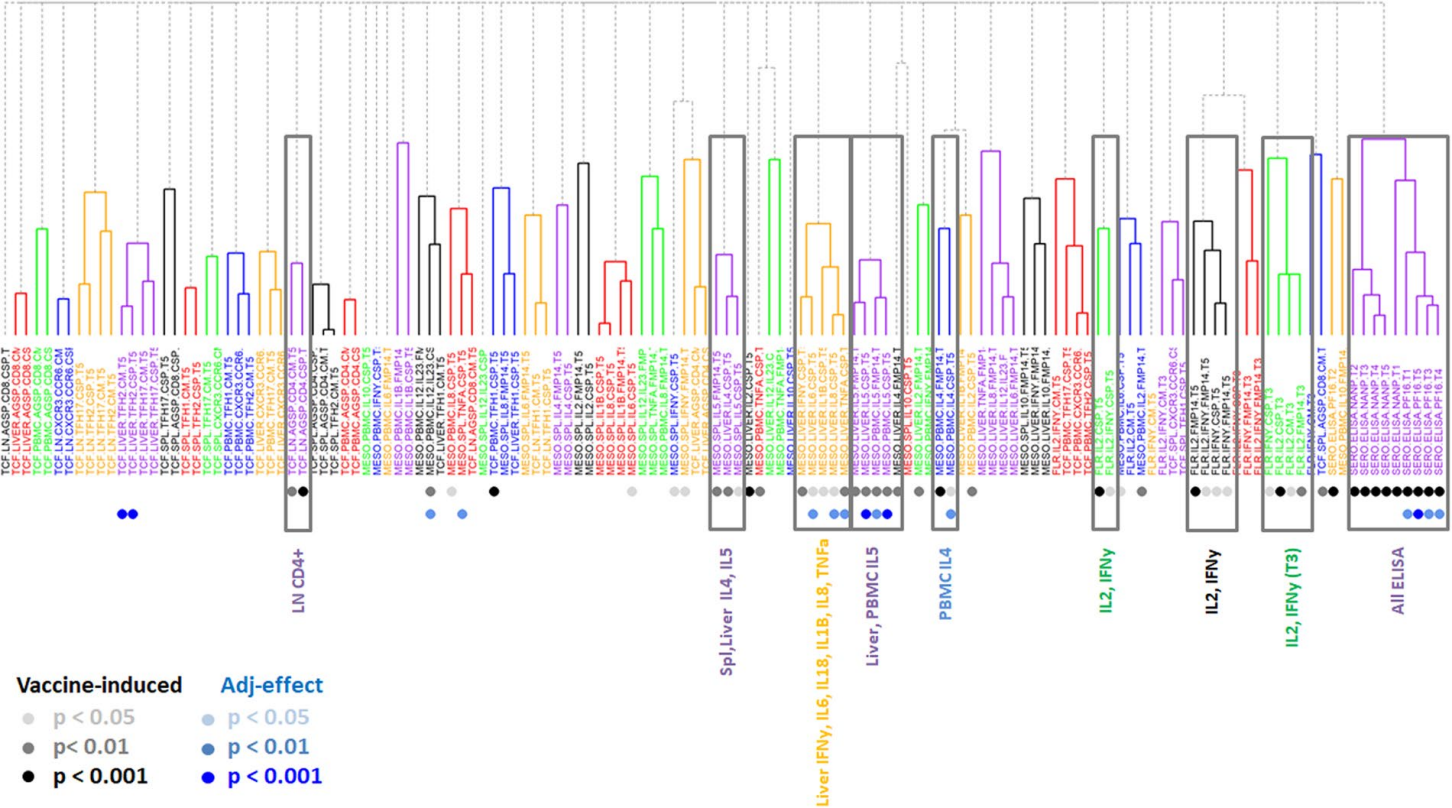
Hierarchical clustering of vaccine-induced immune responses. Hierarchical clustering of immune responses based on their correlation coefficients is shown, colored by immune cluster. Shaded circles below the immune measures indicate statistical significance as a vaccine-induced response or an adjuvant effect. Cluster names are shown, and clusters that predominantly show vaccine-induced measures are highlighted.

Integrating all measurements allowed the analysis of data in regards to which responses were (1) vaccine-induced by comparison with either pre-immune (where available) or adjuvant-saline control animals; (2) sensitive to vaccine dose; and (3) sensitive to the adjuvant formulation (Fig. 2).

We carried out univariate analyses of all immune measures in the study, compared to either their corresponding pre-immune measures or the pooled non-vaccinated control cohort, in order to identify vaccine-induced immune responses. We found vaccine-induced responses in all serological data, as well as in the majority of cytokine responses (IL4, IL5 responses in Ag-stimulated lymphocytes resident in the liver and spleen, as well as IL5 responses in Ag-stimulated PBMCs). Vaccine-induced changes in the frequency of Ag-specific, IL2-producing T cells were statistically significant and higher than for Ag-specific IFN-γ-producing cells. Interestingly, a relatively small number of vaccine-induced responses were observed when analyzing the changes in the T cell subsets (flow cytometric phenotyping of responding cells). These changes could be detected in the frequency of CSP-specific CD4^+^ cells in the lymph nodes, CSP-specific cTFH2 CD4^+^ cells in the liver, and CSP-specific cTFH1 cells in the blood.

We carried out univariate analyses on the vaccine-induced immune responses comparing the pooled adjuvant groups (ALFA *vs*. ALFQ) and the pooled antigen dose groups (low-dose and high-dose). We found 12 immune responses that showed significant differences with respect to adjuvant (p < 0.05 and q < 0.20), but no differences in immune responses with respect to antigen dose. The adjuvant-specific differences included serum antibody response to the CSP C-terminal region at multiple time points, as well as differences in the cytokine response for IL4 and IL5 in both PBMCs and the liver, and for IL6, IL8, and TNFα in the liver.

We carried out a 2-way ANOVA to compare adjuvant and dose conditions for all immune parameters that were classified as vaccine-induced. Among the 12 parameters that showed adjuvant-specific differences in the univariate analysis, 8 of 12 were found to have significant adjuvant effect in the ANOVA (p < 0.05). We did not observe any significant dose effects or adjuvant-dose interaction effects. Supplemental Table S2 provides a summary of the univariate analysis and the ANOVA analysis for the 12 parameters that showed adjuvant-specific differences.

Overall, we found 53 immune measures that we classified as vaccine-induced. We carried a correlation analysis for all vaccine-induced immune measures (Supplemental Fig. S1) followed by hierarchical clustering and found that 10 immune clusters represented almost 80% of the vaccine-induced immune responses. Representative parameters from each of the 10 clusters are shown in Table 1.

**Table 1:** Clusters and representative parameters for vaccine induced responses.

Cluster Name^a^	Assay^b^	Compartment^c^	Phenotype^d^	Representative Parameter^e^
TCF.LN.CD4	T cell flow cytometry	LN	CD4	TCF.ln.AgSp.CD4.CSP.T5
MESO.IL4	Mesoscale	Spl, Liver	IL4, IL5	MESO.liver.IL4.CSP.T5
MESO.IL5		Liver, PBMC	IL5	MESO.liver.IL5.CSP.T5
MESO.Multi		Liver	IFN-y, IL6, IL18, IL1β, IL8, TNFα	MESO.liver.IFNy.CSP.T5
MESO.PBMC.IL4		PBMC	IL4	MESO.pbmc.IL4.FMP14.T5
FLR.IL2	Fluorospot	PBMC	IL2, IL2 + IFN-γ	FLR.IL2.CSP.T5
FLR.IFNy		PBMC	IL2, IL2 + IFN-γ	FLR.IFNy.CSP.T5
FLR.IL2.T3		PBMC	IL2	FLR.IL2.CSP.T3
FLR.IFNy.T3		PBMC	IFN-γ	FLR.IFNy.CSP.T3
SERO.ELISA	ELISA	Serum	NANP, C-term	SERO.ELISA.NANP.T5

^a^Cluster name based on readout (TCF = Flow cytometry; MESO = mesoscale cytokine array; FLR = Fluorospot assay; SERO = serological response measured by ELISA). T3 = time point after last vaccination; T5 = terminal time point (euthanasia);

^b^Readout method used to detect Ag-specific immune responses;

^c^Source of lymphocytes for analysis;

^d^Ag-specific parameter significantly different compared to vaccine controls or pre-immune;

^e^Parameter identifier based on readout method, source of immune cells, significant measurement, time point.

^f^The list of all parameters analyzed as well as the various clusters that are vaccine- or adjuvant-induced is shown in Fig. 2.

### Principal Component Analysis

**Figure 3:**
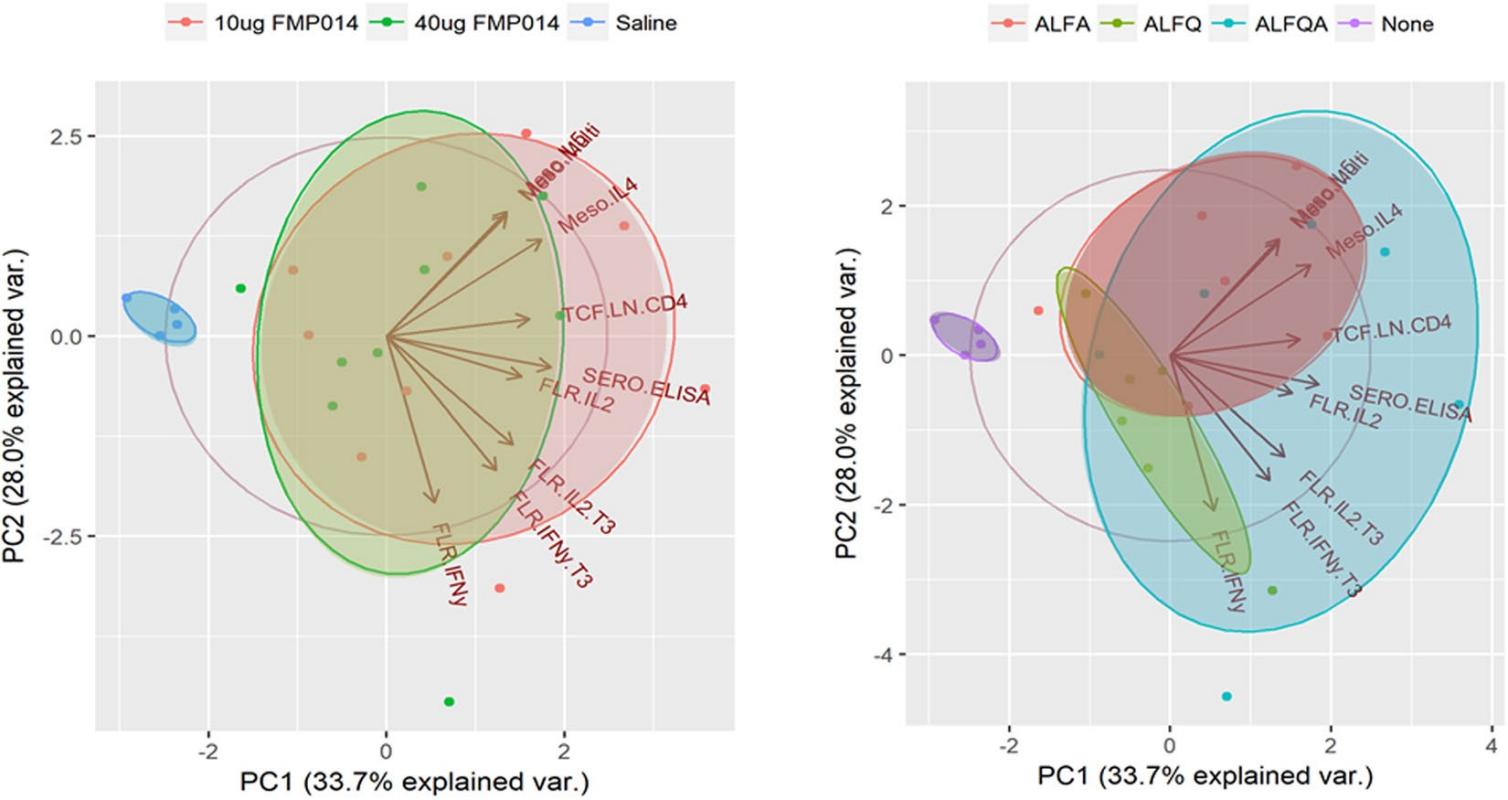
Principal component analysis of vaccine-induced immune responses. The first two principal components (PC1, PC2) are plotted comparing subjects with different antigen doses (left) and different adjuvant conditions (right), compared to non-vaccinated controls. Vectors corresponding to the projection of each immune measure along the two components are shown.

We carried out principal component analyses (PCA) on the representative vaccine-induced immune measures identified by univariate analysis (Fig. 2) (Table 1) and plotted the first two principal components (Fig. 3). The measures for non-vaccinated animals and vaccinated animals formed distinct clusters in the PCA plot, consistent with our classification of these immune measures as vaccine-induced. In agreement with the univariate analysis, the measures for low-dose and high-dose animals showed large overlap in the PCA plot, indicating that there were no significant dose-specific differences in the vaccine-induced immune responses. However, the measures for ALFA and ALFQ animals clustered in distinctly different regions of the PCA plot, indicating a systematic difference in immune responses between these two groups. Interestingly, immune measures for the combination ALFQA animals overlapped considerably with those for both ALFA and ALFQ animals, suggesting both a wider variety of immune responses in the ALFQA condition as well as a commonality in immune responses with both adjuvant groups. We carried out PCA using all 53 vaccine-induced measures (Supplemental Fig. S2) and saw largely similar trends.

### Machine learning

The above analyses confirmed that that ALFA and ALFQ induce distinctly different immune responses when formulated with SAPN. To assess which combination of immune parameters most clearly defines the differences between these two adjuvant conditions, we used a machine-learning method known as the random forest model^17^. We carried out a leave-one-out analysis to train the random forest model and test its accuracy in predicting an animal’s adjuvant condition from its immune response data alone. First, when we used all 53 vaccine-induced immune responses, the resultant model achieved 83% accuracy (kappa = 0.67, p = 0.01) in predicting which animals were given the ALFA- or ALFQ-adjuvanted vaccine. Analyzing for variable importance in the model (Table 2), we found that IL5 responses in the liver and PBMCs, as well as ELISA C-term responses, contributed most to prediction accuracy.

**Table 2:** Variable importance in Random Forest Model using all vaccine-induced parameters.

Assay	Phenotype	Parameter	Weight (53 param model)	Weight (12 param model)
MESO	IL5	MESO.pbmc.IL5.CSP.T5	100.0	100.0
		MESO.liver.IL5.CSP.T5	51.6	36.0
		MESO.pbmc.IL5.FMP14.T5	35.1	28.8
		MESO.liver.IL5.FMP14.T5	32.5	27.3
	IL6	MESO.liver.IL6.CSP.T5	39.6	31.5
	IL4	MESO.pbmc.IL4.CSP.T5	28.0	2.0
	IFNy	MESO.liver.IFNy.CSP.T5	26.0	—
	IL12, IL23	MESO.pbmc.IL12.IL23.CSP.T5	22.9	43.1
FLR	Il2, IFNy	FLR.IL2.IFNy.CSP.T5	52.6	—
		FLR.IL2.IFNy.FMP14.T5	28.0	—
SERO	C-term spec	SERO.ELISA.PF16.T1	60.3	28.5
		SERO.ELISA.PF16.T5	27.6	16.5

To see if we could improve the prediction accuracy, we ran a new random forest model by training it with the 12 immune measures we had identified as having adjuvant-specific differences. The model accuracy improved (92% accuracy, with kappa = 0.83 and p < 0.001) and the variable importance analysis showed good agreement with the random forest model generated using all 53 vaccine-induced measures (Table 2). We also carried out 5-fold and 10-fold internal validation of the model. For the 12-parameter model, we achieved 94% accuracy (kappa = 0.90) in 5-fold internal validation and 97% accuracy (kappa = 0.92) in 10-fold internal validation. For the 53-parameter model, we achieved 85% accuracy (kappa = 0.70) in 5-fold internal validation and 83% accuracy (kappa = 0.65) in 10-fold internal validation. Overall, we found general agreement with all three validation methods, suggesting that these models are able to reliably distinguish between the ALFA and ALFQ adjuvant conditions from vaccine-induced immune responses alone.

**Figure 4:**
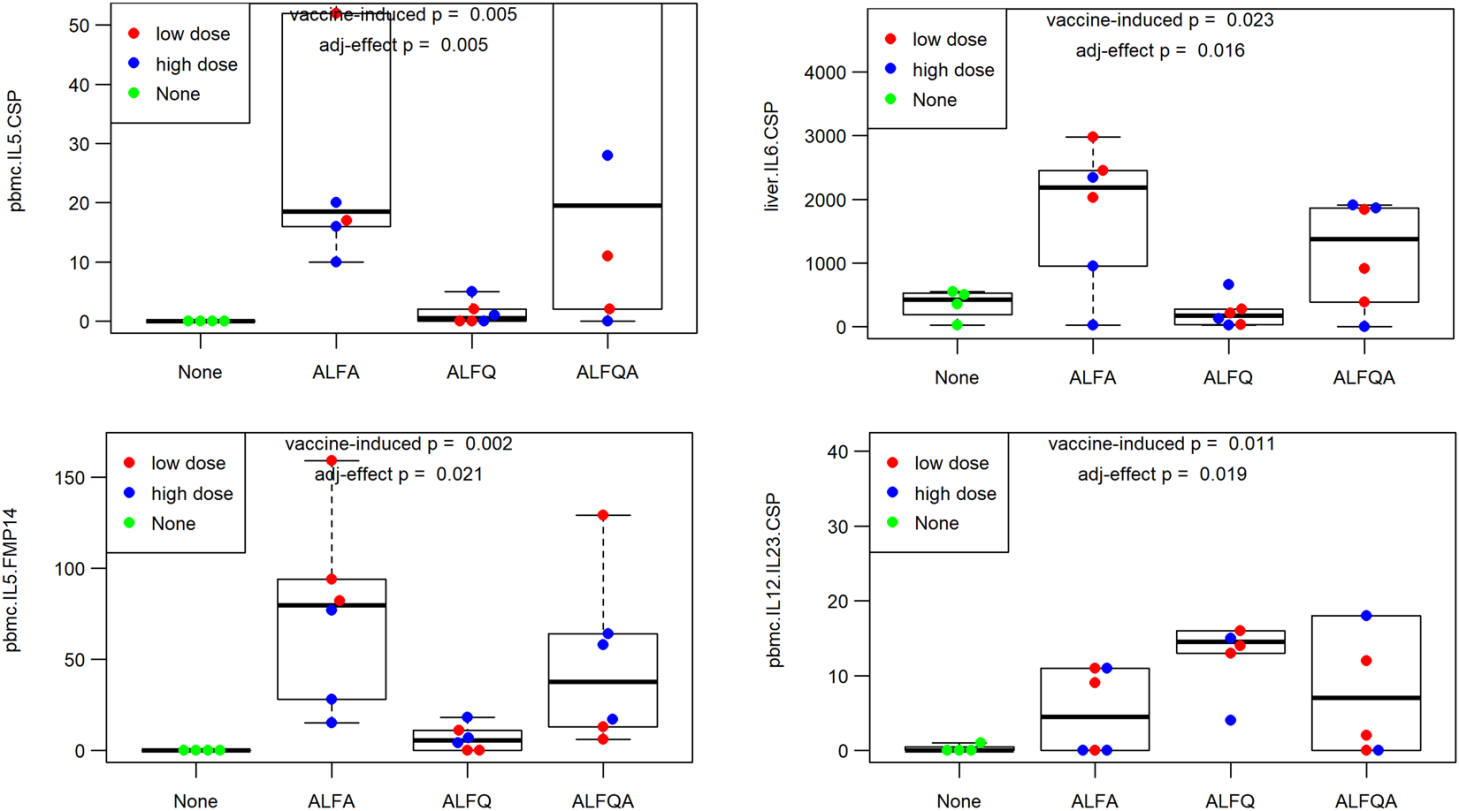
Adjuvant-specific differences in the SAPN-based vaccine. The ALFA-specific response in CSP-specific IL5- and IL6-producing cells (top left and right, respectively). ALFQ-biased and ALFQ-specific responses in CSP C-term-specific ELISA and CSP-specific IL12/IL23p40-producing cells (bottom left and right, respectively).

We identified responses that significantly distinguished ALFA- from ALFQ-adjuvanted vaccines when analyzing the cytokine profile of Ag-specific, liver-resident lymphocyte responses, in particular, of IL1β, IL6, and TNFαl, as well as Ag-specific IL5 responses in liver-resident and PBMC lymphocytes of vaccinated NHPs. Moreover, the frequency of Ag-specific CD4^+^ T cells was also a hallmark feature of recipients of the ALFA-adjuvanted vaccine. In contrast, ALFQ appears to drive higher antibody responses, especially against the C-terminus (PF16) of CSP (Supplemental Fig. S3 and Supplemental Fig. S4). Figure 4 shows representative immune measures for these responses for the three adjuvant conditions.

### Linear regression analysis

Results from both the PCA (Fig. 3) and univariate analysis of immune parameters that distinguish ALFA from ALFQ (Fig. 4) suggest that the ALFQA adjuvant leads to immune responses that have the hallmarks of both ALFA- and ALFQ-specific responses. In order to quantify the relative contribution of ALFA and ALFQ to the ALFQA immune response, we modeled the ALFQA response as a linear combination of ALFA and ALFQ responses.

Starting from the 12 immune parameters that showed adjuvant-specific differences in the univariate analysis (C-terminus-specific antibody titers at all five time points; IL5, IL6, IL8, TNFα responses of CSP-specific liver-resident lymphocytes; IL4, IL5, and IL12/23p40 responses of CSP-specific PBMCs), we carried out a correlation analysis and clustered all parameters that showed a Pearson correlation coefficient of greater than 0.80, and selected a representative parameter for each cluster. This resulted in 8 clusters (Supplemental Table S3). We fit a linear regression model to median values for the 8 representative immune parameters. We fit the following equation, where *M* corresponds to a vector of median values for the 8 immune measures for each adjuvant condition (ALFA, ALFQ, and ALFQA), and *β* corresponds to the weighted contribution of each component adjuvant (ALFA and ALFQ) to the combination adjuvant (ALFQA).

$${M}_{ALFQA}={\beta }_{ALFA}{M}_{ALFA}+{\beta }_{ALFQ}\,{M}_{ALFQ}$$

We found that the immune response measures in the ALFQA condition could be accurately modeled as a linear combination of ALFA and ALFQ responses, obtaining a best fit with *β*_*ALFA*_ = 0.54 and *β*_*ALFQ*_ = 0.49 (p < 10^−4^ and p < 0.01, respectively). We defined three types of adjuvant interactions: 1) antagonistic interactions involving two adjuvants that interfere with and diminish one another’s responses, and can be expressed quantitatively by the condition *β*_*ALFA*_ + *β*_*ALFQ*_ ≪ 1;2) additive interactions involving two adjuvants that ‘average’ their respective immune responses (*β*_*ALFA*_ + *β*_*ALFQ*_ ≈ 1); and 3) synergistic interactions involving two adjuvants that fully combine and/or enhance one another’s immune responses (*β*_*ALFA*_ + *β*_*ALFQ*_ ≫ 1). Within this regime, we found that the ALFQA adjuvant combination is largely additive, with a balanced contribution of both ALFA and ALFQ components.

**Figure 5:**
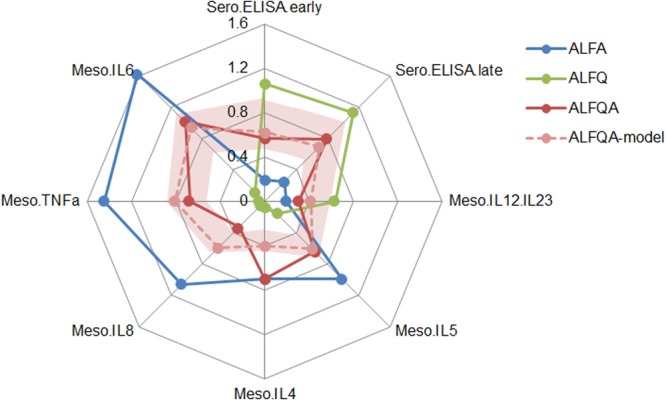
Linear regression model of combination ALFQA adjuvant. Median values for eight representative immune parameters that showed significant differences with respect to adjuvant are displayed in a radar plot using normalized values for ALFA (blue), ALFQ (green), and ALFQA (red). The estimated values based on the linear regression model for ALFQA (pink) is shown along with the 95% confidence interval for the estimated values (shaded pink).

To show how each of the eight immune measures differ between adjuvants ALFA and ALFQ, we plotted the normalized median values in a radar plot (Fig. 5). For the linear regression model, we calculated the expected median values for the immune measures of ALFQA based on the experimental immune measures of ALFA and ALFQ, and compared them with the actual measured values for ALFQA. We found good agreement between the modeled and actual measurements for the ALFQA condition (R^2^ = 0.61), suggesting that a linear model is sufficient to describe the interaction between ALFA and ALFQ in this condition.

We generated a 95% confidence interval for the estimated values for ALFQA based on the linear regression model using a resampling technique (see Methods). Overall, we found that the linear model for the combined ALFQA adjuvant predicted antibody responses at multiple time points, as well as most cytokine measures in the PBMC and liver within the 95% confidence interval, indicating that the combined ALFQA adjuvant can be modeled as a linear combination of ALFQ and ALFA-specific responses. However, we found that two PBMC cytokine measures, IL-4 and IL-5, fell well outside the estimated range. In these cases, the ALFQA adjuvant showed responses very close to the ALFA, suggesting some degree of synergy instead of additivity.

## Discussion

The current report is the first to characterize the impact of adjuvant formulations on the complex immunoprofile of responses in a non-human primate malaria vaccine study, using both adjuvants and vaccine from a clinical development pipeline. The animals had received a high (40 µg) or low dose (10 µg) of FMP014 vaccine formulated with the recently developed adjuvants ALFA, ALFQ, and ALFQA^3,4^. The antigen-specific responses of lymphocytes from multiple anatomical sites (blood, liver, spleen, and draining lymph node) were characterized using cytokine arrays, flow cytometry, and Fluorospot assays. Compiling 120 different measurements from these assays resulted in the definition of distinct immune signatures of these novel adjuvants and a modeling tool capable of predicting the immunoprofile of the vaccine-induced immune response. These signatures can guide the selection of adjuvants, not only for malaria but potentially for vaccines against other pathogens as well by establishing immunological fingerprints of different adjuvants which can be matched with the requirements of vaccine candidates.

Army Liposome Formulations (ALFs) are anionic liposomes^18^ comprised of synthetic phospholipids, cholesterol, monophosphoryl lipids, QS-21 (ALFQ), and/or Alhydrogel (ALFQA and ALFA respectively). These formulations were developed to address the limited availability of adjuvants for clinical use and are based on formulations that have already been extensively used in humans: ALFQ has similarities to AS01^19^, an adjuvant used in Zostervax, which was recently approved by the FDA. AS01 formulations promote the induction of both antibody and cellular responses, and have extensively been tested in the clinic with the RTS,S malaria vaccine, the first licensed malaria vaccine^6,20^. ALFA has similarities to AS04, which is used in Cervarix, an FDA approved HPV vaccine. Alhydrogel-based adjuvants are potent inducers of humoral immune responses. Indeed, testing ALFA with various vaccines against malaria^2,4^, HIV, and heroin^21^ confirmed that this adjuvant induces significant antibody titers. Liposome formulations containing QS21 have been shown to result in strong Th1 responses in mice^3^ and non-human primates (Kaba *et al*., manuscript in preparation). Combining QS21 and Alhydrogel has been reported to increase the potency of the vaccine formulation as well as broaden the immune response to a balanced Th1/Th2 profile in mice^3^. Given the similarities of the ALF formulations to other clinically tested adjuvants, we believe that the defined parameters and modeling approach presented here will be applicable to their clinically used counterparts, and hence provide guidance for the selection of adjuvants for vaccine formulations when immune correlates are known.

Immunoprofiling greatly increases our understanding of the potency of vaccine formulations and holds promise for identifying immune correlates, or at least surrogate markers of, protection. For the present study, we collected 120 different measurements characterizing antigen-specific CD4^+^ (with special emphasis on follicular T helper cells) and CD8^+^ T cell subsets, using specialized flow cytometry^22^, cytokine arrays on culture supernatants of antigen-specifically stimulated lymphocytes, characterization of serological responses regarding fine specificity, and anti-parasite activity in a passive-transfer setting.

Evaluating serological responses, we noticed two major adjuvant-driven differences: (1) CSP-antigen formulated with QS21 (ALFQ and ALFQA) induced significantly more antibodies directed to the C-terminus of the CSP than did that formulated with ALFA; and (2) CSP-antigen formulated with ALFA induced higher antibody titers to the central repeat region of CSP. It is not clear why ALFQ promotes responses to the C-terminal region. Possible explanations are that QS21 has an impact on antigen-presentation to B cells, or that the formulation affects the epitope structure, accessibility, or density and arrangement on the nanoparticle in a manner that alters its relative immunogenicity.

Fluorospot assays that measure the frequency of IFNγ, IL2, and IFNγ /IL2 double-producing cells revealed that all three adjuvants induce IL2 single-producing cells equally, while ALFQ and especially ALFQA induce significantly higher numbers of IFNγ single-producing cells. Vaccination with ALFQ-adjuvanted antigen results in significantly higher frequencies of IFNγ /IL2 double-producing cells. The addition of QS21 appears to favor poly-functional T cells, whereas Alhydrogel promotes the induction of terminally differentiated effector cells. Others have reported that IL2 single-producing cells are linked to the generation of memory cells^23^. This suggests that all three formulations are comparable in their ability to induce T cell memory and terminally differentiated effector T cells. The association of poly-functional T cells with protection has been reported for RTS,S when an Ad35-CSP prime was part of the regimen^24^. Similarly, immunization with adjuvants targeting TLR4 favored the induction of poly-functional T cells in mice protected against challenge with live *Plasmodium* sporozoites^25^. That the ALFQ-adjuvanted vaccine formulation resulted in a higher frequency of poly-functional T cells suggests that this formulation may induce higher protective efficacy.

Data integration and hierarchical clustering demonstrated several findings. First, 53 of the 120 immune measures showed a significant change following vaccination. The 10 immune clusters comprising 38 of the vaccine-induced measures could predict systemic differences between adjuvants, but not dose. Second, classic and commonly used readouts (*i.e*. serology, enumeration of CD4^+^ and CD8^+^ cells producing IFNγ, IL2, and TNFα) missed important differences between the adjuvants. Alhydrogel in ALFA and ALFQA led to significantly higher IL4 and IL5 cytokine responses of antigen-specific lymphocytes at different anatomical sites. This observation is consistent with previous findings that Alhydrogel tends to induce Th2-type immune responses^1^. Third, Alhydrogel induced a pronounced antigen-specific cytokine pattern in the liver that was significantly different from that of ALFQ-induced responses—namely, the induction of pro-inflammatory IL1β, IL6, and IL8 and the Th1 cytokines IFNγ and TNFα. The immunological milieu of the liver tends to down-regulate persistent local immune responses by silencing antigen-specific CD8^+^ T cells through TGFβ and IL10. These immune-dampening cytokines are primarily produced by liver-resident CD4^+^ T cells^26^. Our finding suggests that Alhydrogel-containing vaccine formulations may overcome this tolerance by inducing antigen-specific lymphocytes with pro-inflammatory cytokine profiles.

The current study reveals that immunoprofiling provides the breadth of immune measurements needed for the development of predictive modeling tools. Here, we first performed univariate analyses to identify significant vaccine-specific responses. Next, we carried out correlation analyses followed by hierarchical clustering to identify associations between measurements. Clustering identified 10 groups of correlated immune parameters, or clusters that capture the vaccine-induced responses for the SAPN vaccine candidate. PCA using representative parameters for these immune clusters revealed systematic differences between animals with respect to adjuvant condition, but not vaccine dose. To determine if these parameters could be used to generate an ‘immune signature’ that could clearly define the adjuvant condition, we used a random forest model to create a mathematical model that predicts adjuvant condition from immune data alone. When we used all 53 vaccine-induced parameters to build the model, we achieved an accuracy of 83% for predicting which signature was associated with a specific adjuvant. When we used the 12 parameters that differed between the adjuvants, the predictive accuracy of the model improved to 92%. The random forest model results suggest that these 12 parameters, spread between serological responses such as the ELISA antibody titers, as well as cellular responses such as IL4 and IL6 responses in the liver, are sufficient to discriminate between the ALFA and ALFQ adjuvants. Furthermore, using linear regression modeling, we showed that these parameters were sufficient to quantify the relative contribution of ALFA and ALFQ to the ALFQA combination adjuvant.

In conclusion, this NHP study represents the first comprehensive immunoprofiling effort for three adjuvant formulations that are based on previously described proprietary adjuvants which had not only been tested extensively in humans, but are part of three vaccines licensed for use in humans (Zostervax, Mosquirix, Cervarix). By using a wide range of immune data combined with multivariate analysis and machine learning, we were to identify immune “fingerprints” unique to specific adjuvant formulations and reflect their respective biases towards promoting different types of serological and cellular responses. In upcoming clinical studies with these adjuvant formulations, these immune fingerprints can serve as a guide for defining a more limited selection of accessible human immune parameters that can be used to identify potential correlates of protection. More generally, this approach of applying machine-learning methods to vaccine-induced immune responses can be applied to define complex multi-variate immune signatures that reliably distinguish subjects based on characteristics such as vaccine dose, vaccine regimen, or protection status, and provide insights into the immunological mechanisms that underlie vaccine efficacy.

## Methods

### Study design and samples

Twenty-two non-human primates (NHPs, *Macaca mulatta*) were divided into nine vaccine cohorts and immunized three times in four-week intervals (Supplemental Table S1). Serum and PBMC samples were collected two weeks after each vaccination and cryopreserved. NHPs were euthanized 4–6 weeks after the last immunization; spleens, lymph nodes, liver, and bone marrow were collected; and lymphocytes were prepared and cryopreserved. The research was conducted under an approved animal use protocol in an AAALAC accredited facility, in compliance with the Animal Welfare Act and other federal statutes and regulations relating to animals and experiments involving animals; it also adheres to the principles stated in the *Guide for the Care and Use of Laboratory Animals* (NRC Publication, 2011 edition).

### Immunizations

All vaccines were administered by intramuscular injection into the rectus femoris muscles, with injection sites alternating following each immunization. Serum and blood samples were collected 14 days prior to the first immunization and each subsequent immunization, 35 days after the third immunization, and at the time of euthanasia for tissue collection.

### Mesoscale 10-plex Cytokine U-plex Panel

Cryopreserved pre-immune PBMCs (when available) and post-immune PBMCs (collected at the final time point), splenocytes, lymph node cells, and liver-resident lymphocytes from each NHP were stimulated with a CSP mega-pool representing the FMP014 vaccine peptide pools (15-mer peptides overlapping by 11 AA) at a final concentration of 1 µg/mL for 48 h. A U-PLEX^®^ Biomarker Group 1 (NHP) Assays, SECTOR 10-plex kit (IL1β, IL6, IL8, IL4, IL5, IL10, IL12/IL23p40, IL2, IFNγ, TNFα, Mesoscale Discovery, Gaithersburg, MD) was used to analyze culture supernatants according to the manufacturer’s protocol. Plates were read using a QuickPlex SQ120 plate reader.

### Polychromatic Flow Cytometry Staining

All monoclonal antibodies for cell culture and analysis were purchased from Miltenyi Biotec (San Diego, CA) unless otherwise stated. Cryopreserved pre-immune and final time-point PBMCs, splenocytes, liver-resident lymphocytes, and lymph node cells were cultured for 16 h (37 °C, 5% CO_2_) at a concentration of 2.5 × 10^7^ cells/ml in complete medium (RPMI-1640 with 10% fetal bovine serum, Pen/Strep, L-glutamine, NEAA, Sodium Pyruvate, 2-mercaptoethanol) with CD28 and CD49d (both from BD Biosciences, San Jose, CA) at 1.0 µg/mL, anti-human CCR6-APC (REA190) at 1:10, and CD40 pure (HB14) at 1:100, or in the presence of CSP Megapool (pool representing the *P. falciparum* CSP sequence) at 1.0 µg/mL in addition to the aforementioned. Following stimulation, cells were washed, resuspended, and stained 1:10 with anti-human CD154-biotin (5C8) for 15 min at 4 °C in FACS solution (1.0% fetal bovine serum and 0.1% sodium azide in DPBS). Cells were further incubated 1:5 with anti-biotin microbeads, ultrapure for 15 min at 4 °C. After washing, a pre-titrated and optimized antibody cocktail with fluorochrome-conjugated antibodies [CD3-BV421 (BD Biosciences), CD4-PerCPVio700 (M-T466), CD185-PE-Cyanine7 (MU5UBEE) (eBioscience, San Diego, CA), CD183-VioBrightFITC (REA232), anti-biotin-PE (5C8), and Zombie Aqua Fixable dye (BioLegend, San Diego, CA)] was added, and the cells were incubated for 45 min at 4 °C. After washing in flow buffer, the cells were resuspended in DPBS/0.5% BSA containing 2 mM EDTA, enriched over a magnetic column, and acquired on a MACSQuant Analyzer 10 (Miltenyi Biotec).

The gating strategy is depicted in Supplemental Fig. S5. Lymphocytes were first gated by scatter, then singlets, and finally viability and the lineage marker CD3. Enriched antigen-specific cells were gated by co-expression of CD4 and CD154, followed by the co-expression of CD4 and CXCR5. Within the CD4^+^CXCR5^+^ cells, subsets were further identified—based on CCR6 and CXCR3 expression—as Tfh1, Tfh2, and Tfh17 cells. Enriched antigen-specific CD8^+^ T cells were also analyzed for the expression of CXCR3 and CCR6. The quantitative analysis was performed using FlowJo 10 (Treestar, Ashland, OR).

### ELISA

Assessment of humoral immune responses was performed by ELISA, as previously described^4^. Briefly, ELISA plates were coated with 25 ng of peptide representing either the repeat region (NANP)_6_ or the C-terminus of CSP (PF16). Plates were blocked with 1% casein buffer (ThermoFisher Scientific, Waltham, MA), and tested with serially diluted mouse sera. OD = 1 antibody titers were determined using HRP-labeled goat-anti-mouse IgG (KPL, Gaithersburg, MD) as the secondary antibody and the ABTS substrate (KPL) for detection.

### IL2/IFNγ Fluorospot assay

Antigen-specific interferon (IFN)-γ, interleukin 2 (IL2), and IFNγ/IL2 cytokine-secreting T cells were measured by Fluorospot (U-CyTech Biosciences, Utrecht, Netherlands) following the manufacturer’s instructions. Briefly, activated Fluorospot plates were coated with a solution of IL2- and IFN-γ-coating antibodies and incubated overnight at 4 °C. Stimulating antigens were diluted in culture medium (RPMI-1640 containing 10% fetal bovine serum, Pen/Strep, L-glutamine, NEAA, Sodium Pyruvate, 2-mercaptoethanol). Monkey anti-CD3 mAb (Mabtech Inc., Cincinnati, OH) was used as an internal positive control. Each well was treated with 25 µl of CD28 and CD49d (BD Biosciences, San Diego, CA) cell stimulants, 25 µl of antigen, and 50 µl of cells. Cells were plated at a cell concentration of 2.5 × 10^5^ cells/well. Plates were incubated at 37 °C, 5% CO_2_, 100% humidity for 40 to 48 h. Fluorospot plates were analyzed using the Autoimmun Diagnostica (AID) GmbH Fluorospot reader (Strassberg, Germany) equipped with filters for FITC (excitation 490 nm/emission 510 nm) and Cy3 (excitation 550 nm/emission 570 nm).

### Univariate analysis

To determine which immune responses showed vaccine-induced changes, we carried out univariate analysis for each immune measure. For the serology and Fluorospot data, we had subject-matched pre-immune data and used the paired t-test to calculate statistical significance. For the Mesoscale and T cell flow cytometry data, no pre-immune data were available; therefore, we carried out t-tests between vaccination cohorts pooled by adjuvant (ALFA, ALFQ, and ALFQA) and dose (10 µg and 40 µg). Prior to any t-test, we carried out a Shapiro-Wilks test to determine if the to-be-compared distributions were normally distributed. If both were normally distributed (p < 0.05 by the Shapiro-Wilks test) we applied a Student’s t-test; if either distribution was not normally distributed, we applied the Wilcoxon signed-rank test. After calculating p-values for immune parameters in the data set, we calculated an adjusted p-value using the Benjamini-Hochberg correction^27^. Immune measures in which comparison to either pre-immune or control group data showed a significant difference at p < 0.05 and a false discovery rate of q < 0.20 were classified as vaccine-induced immune responses.

To identify immune measures that differed with respect to adjuvant and antigen dose, we pooled the vaccinated cohorts by adjuvant (ALFA, ALFQ, ALFQA) and antigen dose (10 µg and 40 µg), respectively. We then compared the adjuvant (ALFA *vs*. ALFQ) and antigen dose (10 µg *vs*. 40 µg) as described above, by applying either Student’s t-test or the Wilcoxon signed-rank test following a multiple test correction. Immune measures that showed a significant difference between pooled adjuvant groups or pooled antigen dose groups at p < 0.05 and q < 0.20 were classified as measures showing adjuvant-specific and dose-dependent differences, respectively.

### Multivariate Analysis

Correlation matrices for the data set were generated by calculating the Spearman correlation coefficient between each immune measure and every other immune measure. Subjects with missing data for any pair of immune measures were omitted from that particular correlation calculation. Spearman’s ρ statistic was used to calculate p-values for each correlation estimate. Only correlation coefficients with p < 0.05 were retained for further analysis to ensure that only high-confidence correlations were used in subsequent analyses; all others were set to ‘0’. Hierarchical clustering (R package *hclust* function) was used to group correlated immune measures and to define immune clusters based on a cutoff criterion of having a correlation coefficient of at least 0.40, using the *cutree* function. A dendrogram of the hierarchical cluster was generated using the *A2Rplot* function in R package *addicted*.

Representative immune measures from the ten largest clusters (representing almost 80% of all vaccine-induced immune responses) containing at least one vaccine-induced immune measure were used for additional analyses. Principal component analysis (PCA) was performed on these ten representative immune measures, using the R function *ir.pca* to separate subjects with respect to adjuvant (ALFA, ALFQ, and ALFQA) and antigen dose (10 µg and 40 µg). Using the R package *ggbiplot*, the first two principal components were visualized as two-dimensional PCA plots.

The random forest model was generated using all vaccine-induced immune responses (R *caret* package). We trained the model using the repeated *cv* method, subsampling the data set by 5-fold and resampling ten times. The random forest model was tuned using the *caret* R package. Specifically the number of branches of the tree (*mtry*), the rule for splitting (*gini* or *extratrees*), was adjusted to identify the optimal accuracy and kappa values during internal 10-fold cross validation, repeated 10 times. The *oneSE* method was used to select the optimal model. For both the 53 parameter model and 12 parameter model, the optimal parameter was *mtry* = 2 and splitrule = *gini*. To test the predictive accuracy of the random forest modeling approach, we carried out a leave-one-out analysis, where one subject was removed from the data set, after which the model was trained on the remaining subjects and then used to predict the adjuvant condition of the excluded subject based on its immune data. We performed this for all subjects in the data set, and calculated both the accuracy and kappa value of the prediction model. We used the *varImp* function to determine the variable importance for each generated model, and reported the average variable importance across all models to assess the relative importance of each vaccine-induced immune measure to predicting the adjuvant condition.

We used linear regression modeling to determine the relative contributions of ALFA and ALFQ adjuvants to the combined ALFQA adjuvant condition. We carried out correlation analysis of all 12 immune parameters that showed adjuvant-specific differences in the univariate analysis, and generated clusters for immune measures that showed a Pearson correlation coefficient > 0.80 (Supplemental Table S3). From each cluster, the parameter that showed the greatest effect-size in the univariate analysis between the ALFA and ALFQ conditions was selected as the representative parameter for that group. We found that all eight parameters were normally distributed in at least one of the adjuvant cohorts using the Shapiro test (p > 0.05). We defined immune responses for ALFA and ALFQ as vectors of median values of the pooled ALFA and ALFQ vaccinated cohorts, respectively, for the eight representative immune measures. We then fitted the median ALFQA response across those same 12 measures (*M*_*ALFQA*_) as the sum of the ALFA and ALFQ responses (*M*_*ALFA*_ and *M*_*ALFQ*_), weighted by *β*_*ALFA*_ and *β*_*ALFQ*_, respectively. Because we sought to express the ALFQA response as entirely a function of the ALFQ and ALFA responses, we suppressed the constant term from the equation.

$${M}_{ALFQA}={\beta }_{ALFA}{M}_{ALFA}+{\beta }_{ALFQ}\,{M}_{ALFQ}$$

The coefficients *β*_*ALFA*_ and *β*_*ALFQ*_ reflect the relative contributions of ALFA and ALFQ, respectively, to the ALFQA adjuvant condition. We classified the interaction of these two adjuvants by its relative contribution to the combined adjuvant condition, using the following criteria:

$${\beta }_{ALFA}+{\beta }_{ALFQ}\ll 1\,({\rm{antagonistic}})$$

$${\beta }_{ALFA}+{\beta }_{ALFQ}\approx 1\,({\rm{additive}})$$

$${\beta }_{ALFA}+{\beta }_{ALFQ}\gg 1\,({\rm{synergistic}})$$

In order to generate an estimate of the 95% confidence interval for the linear regression model, we carried out a boot-strapping approach where we re-sampled the ALFA and ALFQ data set, with replacement, 1000 times, and generated a linear regression model for each re-sampled data set. We modeled the ALFQA response based on the coefficients and the median ALFA and ALFQ values generated from each of the re-sampled data sets, and report the 2.5 percentile and 97.5 percentile values for each immune parameter.

All the analysis scripts used in this study were written in R and are available freely for download at https://github.com/BHSAI/immstat. Please contact the corresponding author for technical support or further information.

## Electronic supplementary material

Supplementary Figures and Tables
