# Supplementary material for: Identification of Immune Signatures of Novel Adjuvant Formulations Using Machine Learning
Source: Sci Rep. 2018 Nov 30;8:17508. doi: 10.1038/s41598-018-35452-x (PMC6269591; doi:10.1038/s41598-018-35452-x)
Supplement: Supplementary file 1 — Supplementary Figures and Tables [file 41598_2018_35452_MOESM1_ESM.pdf]

Identification of Immune Signatures of Novel Adjuvant Formulations Using Machine Learning

Sidhartha Chaudhury[1*], Elizabeth H Duncan[2], Tanmaya Atre[2], Casey K Storme[2], Kevin Beck[3], Stephen A. Kaba[2], David E Lanar[2], Elke S Bergmann-Leitner[2*]

1. Biotechnology High Performance Computing Software Applications Institute, Telemedicine and Advanced Technology Research Center, U.S. Army Medical Research and Materiel Command, Fort Detrick, MD, USA
2. Malaria Vaccine Branch, US Military Malaria Research Program, Walter Reed Army Institute of Research, Silver Spring, MD, USA
3. Miltenyi Biotec Inc., San Diego, CA, USA

*Corresponding authors:

Dr. Elke S. Bergmann-Leitner, email: elke.s.bergmann-leitner.civ@mail.mil

Dr. Sidhartha Chaudhury, email: sidhartha.chaudhury.civ@mail.mil

SUPPLEMENTARY MATERIAL

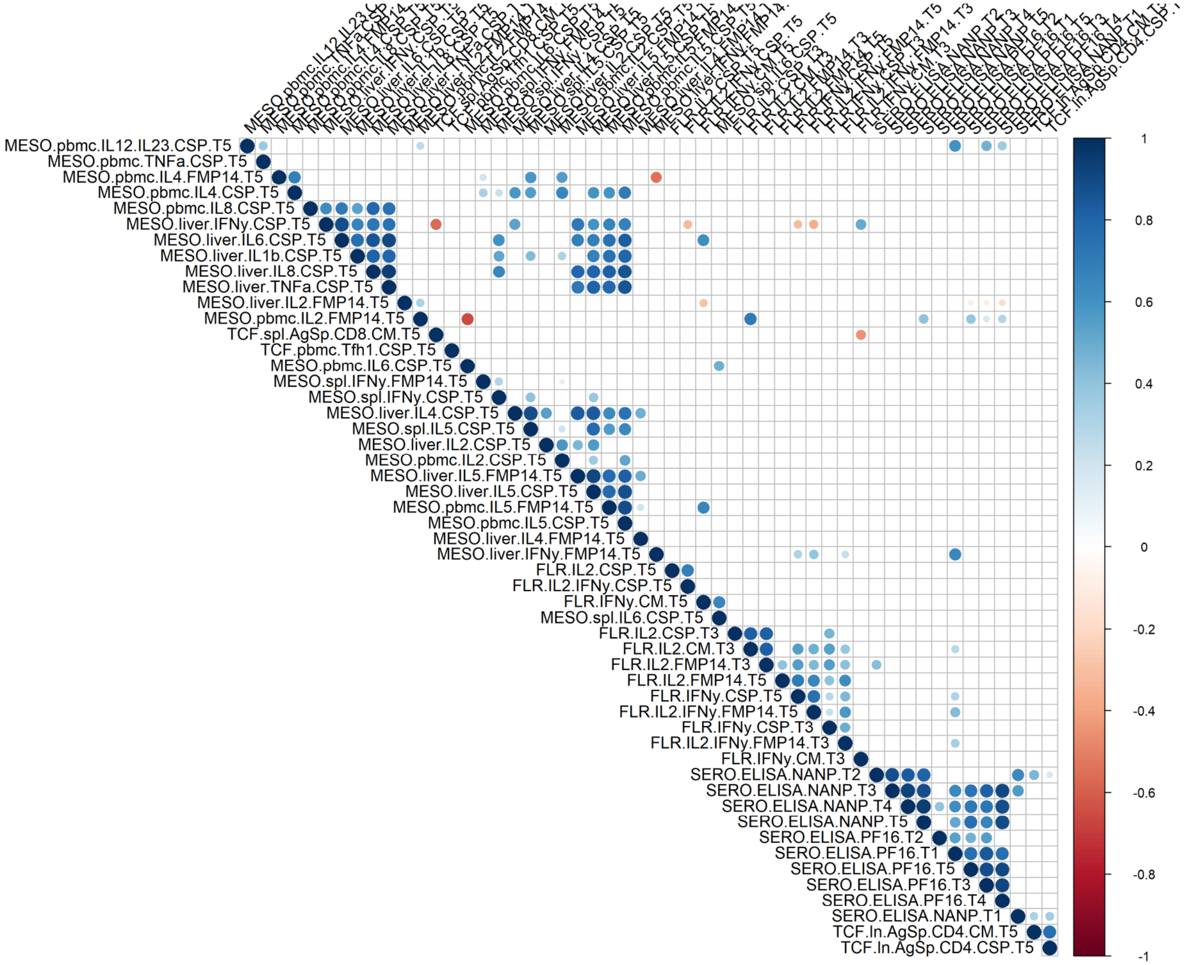

Supplementary Figure S1. Correlation matrix for all 53 vaccine-induced immune responses is shown using the Spearman's test. Positive correlations are shown in blue, negative correlations in red. All correlations shown are statistically significant ($p < 0.05$).

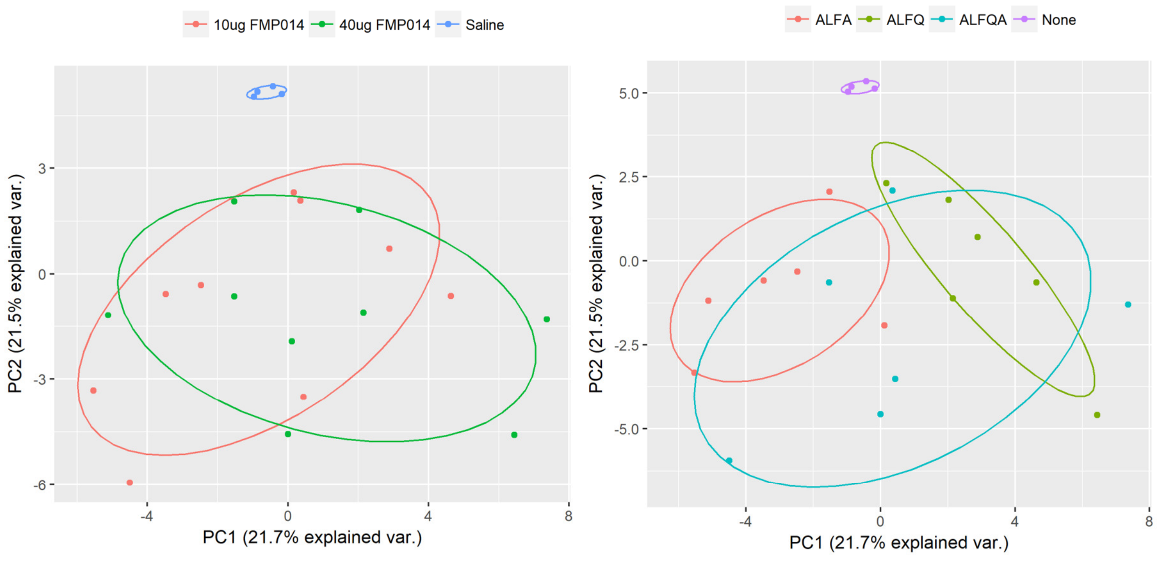

Supplementary Figure S2. PCA plots using all 53 vaccine-induced immune responses. The first two principal components (PC1, PC2) are plotted comparing subjects with different antigen doses (left) and different adjuvant conditions (right), compared to non-vaccinated controls.

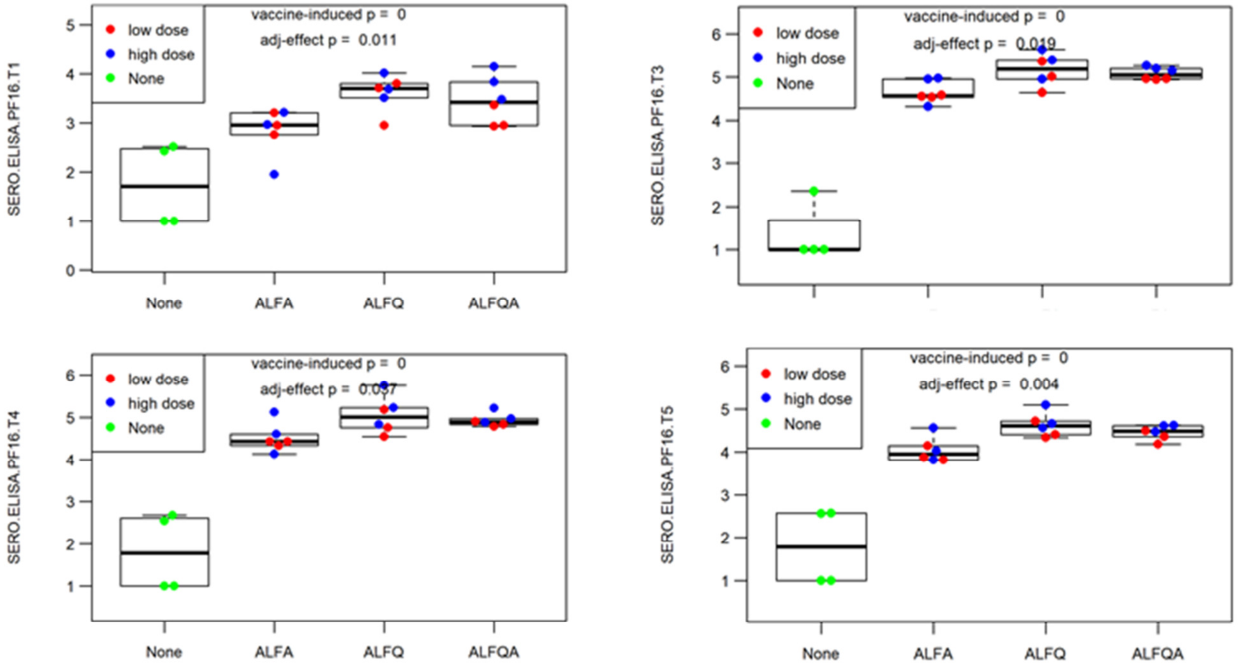

Supplemental Figure S3. ELISA results for the C-terminal region of CSP (PF-16) for the ALFA, ALFQ, and ALFQA adjuvant conditions under low-dose (red) and high-dose (blue) conditions.

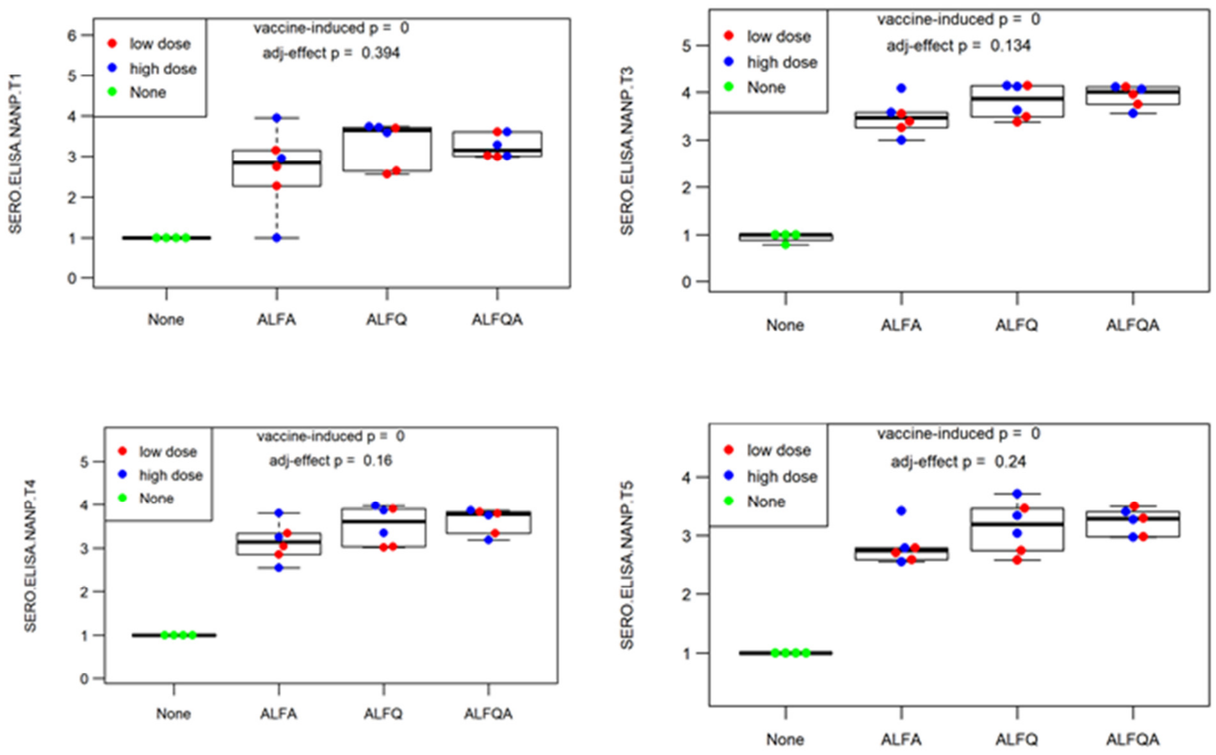

Supplementary Figure S4. ELISA results for the repeat region of CSP (NANP) for the ALFA, ALFQ, and ALFQA adjuvant conditions under low-dose (red) and high-dose (blue) conditions.

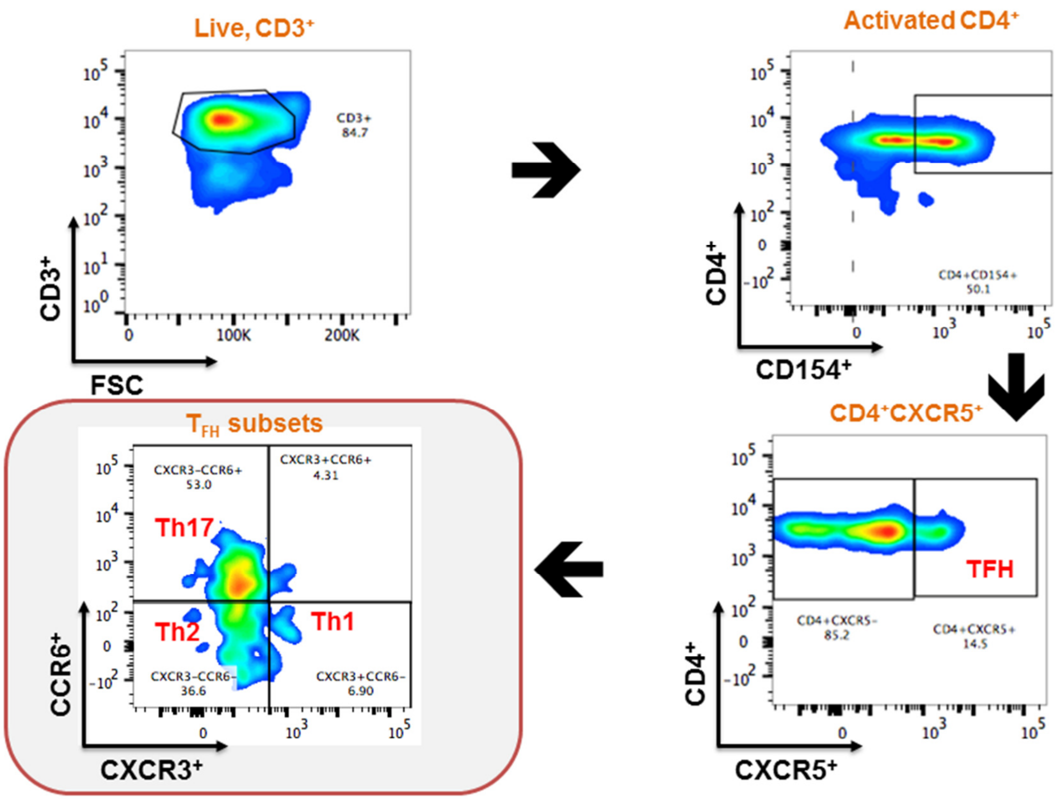

Supplemental Figure S5. Gating strategy for flow cytometric analysis. After antigen stimulation, cells were gated based on viability (not shown here) and expression of CD3. This population was then further gated based on the expression of activation marker CD154 (cells are considered "antigen-specific") and lineage marker CD4. Antigen-specific $CD4^{+}CXCR5^{+}$ cells were then analyzed for the concomitant expression of CXCR3 (Tfh1) and CCR6 (Tfh17).

Supplemental Table S1

Cohort	Type	N	Vaccine Dose	Adjuvant
Low Dose, ALFA	Vaccine	3	10 µg FMP14	ALFA
Low Dose, ALFQ	Vaccine	3	10 µg FMP14	ALFQ
Low Dose, ALFQ+A	Vaccine	3	10 µg FMP14	ALFQ+A
High Dose, ALFA	Vaccine	3	40 µg FMP14	ALFA
High Dose, ALFQ	Vaccine	3	40 µg FMP14	ALFQ
High Dose, ALFQ+A	Vaccine	3	40 µg FMP14	ALFQ+A
ALFA only	Control	1	Saline	ALFA
ALFQ only	Control	1	Saline	ALFQ
ALFQ+A only	Control	1	Saline	ALFQ+A

Supplemental Table S2

		Univariate Analysis						ANOVA		
		Vaccine-induced		*Adjuvant-effect*		*Dose-effect*		*Adjuvant*	*Dose*	*Adjuvant*Dose*
**Parameter**	**Cluster ID**	**p-value**	**q-value**	**p-value**	**q-value**	**p-value**	**q-value**	**p-value**	**p-value**	**p-value**
SERO.ELISA.PF16.T5	1	1.9E-08	1.9E-08	0.004	0.009	0.158	0.245	4.0E-06	0.423	0.990
SERO.ELISA.PF16.T1	1	2.9E-07	2.9E-07	0.011	0.022	0.361	0.361	7.1E-04	0.485	0.510
SERO.ELISA.PF16.T3	1	5.5E-08	5.5E-08	0.019	0.039	0.118	0.236	8.9E-09	0.361	0.980
SERO.ELISA.PF16.T4	1	1.1E-08	1.1E-08	0.037	0.074	0.133	0.265	3.5E-05	0.426	0.945
MESO.pbmc.IL5.CSP.T5	17	0.005	0.027	0.005	0.183	0.722	0.985	0.038	0.319	0.236
MESO.liver.IL5.CSP.T5	17	0.005	0.027	0.008	0.183	0.625	0.915	0.132	0.961	0.984
MESO.pbmc.IL5.FMP14.T5	17	0.002	0.020	0.021	0.183	1.000	1.000	0.023	0.216	0.184
MESO.liver.IL6.CSP.T5	18	0.024	0.070	0.016	0.183	0.472	0.915	0.005	0.289	0.110
MESO.liver.IL8.CSP.T5	18	0.026	0.071	0.018	0.183	1.000	1.000	0.077	0.910	0.950
MESO.liver.TNFa.CSP.T5	18	0.005	0.027	0.028	0.187	0.592	0.915	0.031	0.293	0.365
MESO.pbmc.IL12.IL23.CSP.T5	34	0.011	0.045	0.019	0.183	0.688	0.960	0.290	0.207	0.354
MESO.pbmc.IL4.CSP.T5	36	0.018	0.061	0.028	0.187	0.883	0.994	0.316	0.606	0.536

Supplemental Table S3

**Cluster name**	**Parameter**
Sero.ELISA.early	ELISA.PF16.T1
Sero.ELISA.late	ELISA.PF16.T3
	ELISA.PF16.T4
	ELISA.PF16.T5
Meso.IL12.IL23	pmbc.IL12.IL23.CSP
Meso.IL8	liver.IL8.CSP
Meso.IL6	liver.IL6.CSP
Meso.TNFa	liver.TNFa.CSP
Meso.IL4	pmbc.IL4.CSP
Meso.IL5	pbmc.IL5.CSP
